# Sensing of Scent, Fragrance, Smell, and Odor Emissions from Biota Sources

**DOI:** 10.3390/s140406567

**Published:** 2014-04-09

**Authors:** Ki-Hyun Kim

**Affiliations:** Department of Civil & Environmental Engineering, Hanyang University, Seoul 133-791, Korea; E-Mail: kkim61@hanyang.ac.kr; Tel.: +82-2-2200-2325; Fax: +82-2-2200-1945

## 1. Introduction

People encounter enormous numbers of chemicals present in the outdoor atmosphere and/or in the various facilities they use daily. Despite such diversity, not many of them have necessarily the potential to draw human's nasal attraction if their perception thresholds are in general not sufficiently low enough, regardless of abundance. In this sense, many types of scents, musks, fragrances, smells, odors, and pheromones are unique enough to draw a great deal of attention mainly by their presence at or near threshold levels which are far lower than those of common chemicals with poor odorant characteristics. It is known that most of the diverse characters of odor-related ingredients or expressions are commonly produced from various biota sources present in the biosphere, e.g., fauna, flora, bacteria, fruits, flowers, trees, meats, fresh/decaying foods, *etc*.

In light of the environmental significance of the various odor types characterizing certain odorous events, it is crucially important to be able to describe, both qualitatively and quantitatively, the concentration levels and/or relative composition of both major and minor components giving rise to such odorous conditions. Despite many advances achieved over the past decades in the sensing and instrumental techniques for odor quantitation, it still remains of utmost importance to expand our knowledge on the exact nature of various odor types and improve our odor detection abilities.

## 2. Discussion

The works described in this Special Issue (SI) are thus aimed at covering the topic of collaborative subjects on the application of detection techniques for various odor-related targets which we typically encounter in our everyday livelihood by mainly focusing on the following subjects: (1) sampling techniques for odor, fragrance, and related components; (2) olfactometry; (3) electronic noses; (4) advanced instrumentation (e.g., combination of thermal desorption with GC-MS or MS-MS, GC-GC, *etc.*); and (5) all other available or emerging tools for odor sensing.

A total of 15 articles were finally published in this special issue to cover the topic of sensing of various odorants and odor-related phenomena with the aid of diverse instrumental configurations. The results of this SI can now be classified into several subcategories mainly based on the specific approaches employed by each team of authors. Thus, each of the 15 papers can be classified into one or more specific categories and listed in the appropriate references section. As this SI pursues the introduction of a whole variety of odor detection techniques, it thus offers a nice chance to connect bridges to the world of diverse odor phenomena that exert impacts on our cognition and emotion.

### 2.1. Sampling Techniques

Two papers from our SI deal with issues covering advances in sampling techniques. Pravin *et al.* [1] described how the simultaneous sampling of flow and odorants by crustaceans can aid searches within a turbulent plume. In addition, Young *et al.* [2] introduced the application of receiver operating characteristic (ROC) curves based on different sampling and detection approaches.

### 2.2. Olfactometry

Three of our SI articles cover studies on olfactometry issues of odorants using PTR MS (Hansen *et al.* [3]), analysis of feedstuffs and animal nutrition (Campagnoli *et al.* [4]), and GC-based chemical characterization (Brattoli *et al.* [5]).

### 2.3. Electronic Noses

We received the highest number of papers (four) in this section. Chiu and Tang [6] reviewed a chemiresistive sensor-integrated electronic nose. Fujioka *et al.* [7] introduced an E-nose to discriminate the volatiles from fresh mushrooms. Moreover, Dymerski *et al.* [8] focused on quality evaluation of agricultural distillates. Finally, Wilson *et al.* [9] summarized detection methods for off-flavor in catfish.

### 2.4. Advanced Instrumentation

In this section, we focused on the combination of thermal desorption and GC-MS method for detecting volatiles and odorants. Kim *et al.* [10] provided the method to carry out a quantitative analysis of fragrance and odorants from strawberries. Pandey *et al.* [11] introduced methods to measure major odorants released as urinary volatiles. Covington *et al.* [12] introduced a novel tool for diagnosing bile acid diarrhoea. Paul and Park [13] identified volatiles produced by *Cladosporium cladosporioides* CL-1, a fungal biocontrol agent.

### 2.5. Emerging Techniques

Zarzo [14] dealt with a fresh scent in perfumery to find correlations between perceptual freshness and substantivity. Finally, Soso *et al.* [15] presented a unique review on the chemical and sensory characterization of scent-markings in large wild mammals.
